# Sources of individual variability in a pragmatic reference game: Effects of logical reasoning and Theory of Mind

**DOI:** 10.1371/journal.pone.0339899

**Published:** 2026-02-19

**Authors:** Alexandra Mayn, Vera Demberg

**Affiliations:** 1 Department of Language Science and Technology, Saarland University, Saarbrücken, Germany; 2 Department of Computer Science, Saarland University, Saarbrücken, Germany; CNRS: Centre National de la Recherche Scientifique, FRANCE

## Abstract

While in theory people are expected to adhere to rational communicative principles, a growing body of work shows that people vary widely in their tendency to draw pragmatic inferences. It has been suggested that these differences may, in part, stem from depth of reasoning: Previous work has shown that individual participants’ response patterns in a pragmatic reference game are predicted by three probabilistic pragmatic models of different reasoning depth. However, those models are agnostic to the cognitive traits which underlie those differences. In this study, we systematically investigate sources of individual variation in a pragmatic reference game, where participants are required to draw ad-hoc implicatures of various complexity. We relate the observed variability in reference game performance to cognitive traits, specifically logical reasoning ability, working memory and Theory of Mind, as well as to the strategies reported by participants. We find a positive effect of logical reasoning and Theory of Mind on pragmatic inference. We do not find evidence for an effect of working memory.

## Introduction

The cooperative principle, formalized by the philosopher of language Paul Grice [1], states that communicators should behave cooperatively and adhere to Maxims of Conversation, such as Maxim of Quantity, which requires the speaker to be as informative as is warranted by the situation. Listeners are then expected to derive inferences based on the assumption that the speaker is behaving in accordance with Grice’s rules. For example, upon hearing a guest say “It’s cold in here”, the listener might infer that the guest is politely asking her to turn on the heating or close the window as opposed to merely making a statement about room temperature [2].

While Gricean principles decribe the ideal pragmatic communicator, there is a growing body of work showing that, in practice, people differ vastly in their tendency to derive pragmatic inferences and that some people tend to consistently favor literal interpretations. For example, Heyman & Schaeken [3] identified three groups of responders in a scalar implicature derivation task: consistently pragmatic, consistently literal and inconsistent. Variability has also been observed in sensitivity to context when deriving inferences [4], in metaphor [5] and indirect request [2] comprehension, and in atypicality inferences [6].

Probabilistic pragmatic models, such as the Rational Speech Act framework [7] and closely related Iterated Best Response models [8], aim to formalize Gricean principles and make quantitative predictions for various pragmatic phenomena. In these models, a listener and a speaker recursively reason about each other’s intent, as well as potential alternative meanings and utterances, to arrive at an interpetation. Such models have been shown to provide a close fit to population-level data for various phenomena, including scalar implicature and speaker knowledge [9], hyperbole [10], politeness [11] and rational overspecification [12]. These models have mostly been fit to population-level data, averaging over participants and disregarding differences in the tendency to draw pragmatic inferences.

Franke & Degen [13] (henceforth, F&D) showed that individual differences in participants’ performance on a pragmatic reference game can be explained by positing three probabilistic pragmatic models of different reasoning depth. They conducted an experiment where participants were asked to reason about potentially ambiguous messages allegedly sent by a participant of an earlier study, and showed that participants naturally fell into groups whose performance lined up with the predictions of three probabilistic models for listeners of three different reasoning depths, from the simplest literal model to a pragmatic Gricean model.

To better understand the nature of the observed individual variability and to be able to build more plausible cognitive models of pragmatic processing, it is valuable to investigate factors which drive this variability. While F&D showed that a model where each participant’s data was captured by one of the three probabilistic pragmatic models of different complexity was able to better account for the data than a homogeneous model which assumed that all participants had the same reasoning depth, their model is agnostic about the factors which underlie these differences in pragmatic performance.

The current work adds to the growing body of evidence suggesting that individual differences in pragmatic reasoning are systematic and related to cognitive traits. We systematically investigate the nature of individual variation in the pragmatic reference game and relate it to individual differences in three cognitive traits: working memory capacity, logical reasoning ability, and Theory of Mind. We replicate the variability in the reference game observed by F&D on a much larger sample size (254 participants for the reference game, 167 for the individual differences analysis, as opposed to 51 participants in F&D) and using a different set of stimuli, which has been shown to be less susceptible to biases than the original stimuli [14]. In addition to the reference game, we collect a battery of individual differences, with two measures per construct of interest – logical reasoning ability, working memory and Theory of Mind – in order to ensure robustness of the obtained result. Moreover, we ask participants to report their strategy and inspect the relationship of the reported strategies to groups identified based on performance.

Our analyses reveal a positive effect of logical reasoning ability. In addition to the main effect, we find an interaction with condition, suggesting that logical reasoning ability differentially affects performance on implicatures of different complexity. In addition, we find a positive effect of Theory of Mind, whereby participants with higher mentalizing skills are more likely to draw implicatures. We do not find evidence for an effect of working memory.

## Background

### Reference game

Our task of interest is a pragmatic reference game, a signaling game which has been repeatedly used to verify predictions of probabilistic pragmatic models (e.g., [7,15,16]). It involves a speaker sending a message to refer to one of the objects in the shared view and a listener trying to identify the intended referent based on the message.

The task is a replication of the comprehension experiment (Experiment 1) of F&D, with two modifications. First, instead of using the stimuli from F&D (pictures of monsters and robots with accessories), we opted for abstract stimuli (shapes and colors) since those were shown by Mayn & Demberg [14] to be less susceptible to biases leading participants to select the target due to superficial similarity as opposed to successful reasoning. Secondly, at the end of the task, we elicited participants’ reasoning strategies in order to identify participants who misunderstood the task and to gain insight into whether participants’ reported strategies reflect the assumed reasoning or whether some participants used a different strategy. Strategy elicitation also allowed us to gain more insight into participants’ performance on the task and monitor possible biases in the stimuli. We explain strategy elicitation and annotation in more detail later.

On each experimental trial, participants saw three objects on the display. The objects differed along two dimensions – shapes (triangle, square and circle) and colors (red, green and blue). Participants also saw a message that they were told had been sent by the previous participant, which was either a color or a shape, in order to get them to pick out one of the three objects. Participants were also told that only two of the three shapes and two of the three colors were available to the previous participant to send as messages (square and blue were not available; we’ll call them *inexpressible features*), and the available messages were always displayed at the bottom of the screen.

**Fig 1:**
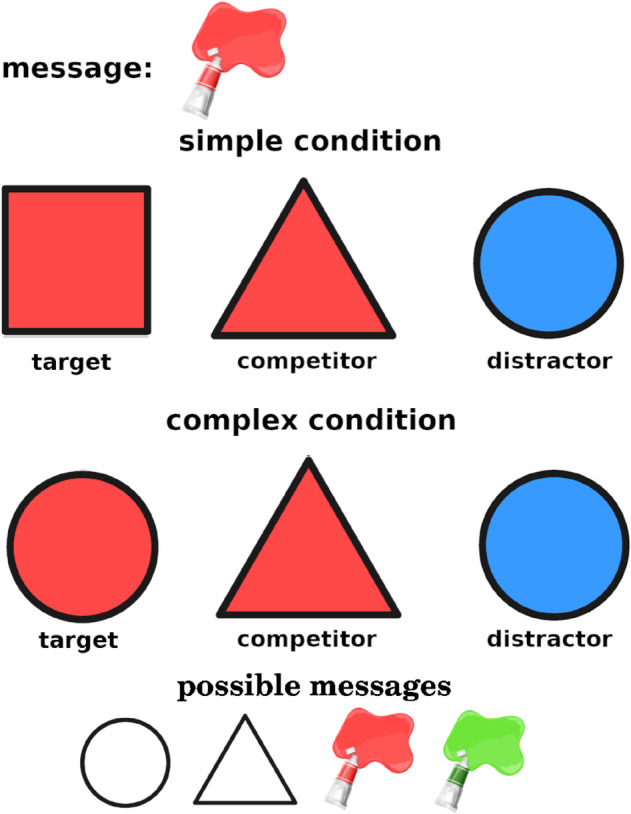
An example of a simple and a complex reference game trial.

In the beginning of the task, participants completed 4 practice trials which took the speaker’s perspective: participants had to select a message to refer to one of three objects on the screen, which was highlighted. Then the actual task began, which consisted of 66 trials, of which 24 were critical and 42 were fillers, distributed as follows:

Half of the critical trials were *simple implicature trials* (top panel of Fig 1). On those trials, the target contained the sampled message and the inexpressible feature along the other dimension. For instance, if the sampled message was the color red, the target would be a red square, since “square" is not an available message. The competitor was composed of the message and an expressible feature along the other dimension. Continuing with our example, the competitor could be a red triangle. Finally, the distractor was composed of any two features not present in the target or competitor. In our example, it could be a blue circle.

On complex implicature trials (bottom panel of Fig 1), the target contained the message along with an expressible feature along the other dimension. So if the sampled message was the color red, the target could be a red circle. The competitor contained the message and the other expressible feature along the other dimension. In this example, the competitor would be a red triangle. The distractor combined the other target feature not denoted by the sampled message with the inexpressible feature along the other dimension. In our example, the distractor would be a blue circle.

The complex implicatures are predicted to be more difficult than the simple ones since a probabilistic Bayesian model can be defined which can solve the simple but not the complex implicatures. We will explain this in more detail when we introduce formal reasoning types. The experimental results of F&D show that the complex condition is indeed more difficult than the simple one. This also holds for the abstract stimuli [14].

Of the 42 filler trials in the study, 24 were identical to the critical trials, except the target was the (6) competitor or (6) distractor from the simple condition, or the (12) competitor from the complex condition, unambiguous given the message. For example, a filler trial would have a display identical to the top panel of Fig 1, except the target would be the red triangle, and the (unambiguous) message would be “triangle”. Of the remaining 18 trials, 9 were completely unambiguous, meaning that they contained each shape and each color only once, and 9 were completely ambiguous, where two of the three objects were completely ambiguous and the message was equally likely to refer to both of them. Ambiguous trials were included as the random baseline, and unambiguous fillers served for determining exclusion. Participants who had accuracy below 80% on the unambiguous trials were excluded from analysis.

### Elicitation of reasoning strategies

After participants completed the 66 reference game trials, they were shown one simple and one complex item again, in that order. While the order of other items was randomized for every participant, the two items which were shown to participants were kept the same for all participants. On those two trials, once participants had made their choice, a red box appeared around their chosen object, a textbox popped up and participants were asked to briefly explain their decision.

The purpose of this was twofold: On the one hand, it helped us get additional insight into the way participants performed the task. We expected the different reasoning strategies corresponding to different reasoning depths hypothesized by F&D to be reflected in the provided explanations. On the other hand, this allowed us to identify participants who might have misunderstood the instructions and therefore needed to be excluded.

Two annotators, one of whom was blind to the purpose of the study, annotated participants’ explanations using an annotation scheme based on Mayn & Demberg [14]. Inter-annotator agreement was substantial, Cohen’s κ = 0.76, 95% CI [0.72, 0.80]. All disagreements were resolved jointly: both annotators made a case for their label, and if agreement could not be reached after that, the explanation was labeled *unclear*.

Example responses for all annotation tags are presented in Table 1.

**Table 1:** Annotation tags used to label participants’ reported reasoning strategies with examples.

Annotation tag	Subtag	Example
correct_reasoning		The participant could have chosen red to uniquely determine the other triangle, but didn’t, indicating it must be the blue triangle.
guess		It’s a 50/50 guess between the two triangles.
guess	screenloc	There are two triangles, and there’s no way for me to know which one is correct, so I chose the first one.
guess	variability	I knew it would be red and the last image was a triangle, so I tried a circle this time.
other_reason	salience	I feel like red is more likely as it’s a brighter colour.
other_reason	preference	[I picked the blue triangle and not the red one] because I like blue more than red.
other_reason	visual_resemblance	The image must be red and the paint splodge looks more like a circle.
other_reason	multiturn	I think red images are shown more often so I have a better chance of being right if I choose the red triangle.
other_reason	other	There is no answer option for the blue circle, so I would go for it if I were the previous participant (as a reason to select the distractor).
odd_one_out		Because it was the only shape that was not red.
misunderstood_instr		Because the color blue is not a choice therefore it must be the green or red shape.
unclear		Because it felt right.
mistake/changed_mind		I meant to select the blue one. I clicked on the red one by accident.

Responses which pointed to correct counterfactual reasoning were labeled *correct_reasoning*. An example for the top panel of Fig 1 would be “If the previous participant had meant to refer to the red triangle, they could have used the triangle message. Since they didn’t, they must have wanted me to pick the square".

Responses which suggested guessing, such as “There are two red objects, so it’s 50/50 between them", were labeled *guess*. There were two other subtypes of *guess* responses. Responses which reported deciding between two objects based on their position on the screen (e.g., picking the first one or the middle one) were additionally assigned the subtag *screenloc*. Explanations which reported picking one of the two objects “to mix things up" since the participant had chosen the other shape or color more often in the past were assigned the subtag *variability*.

The *other_reason* tag was used when an explanation revealed a different strategy from correct reasoning and guessing, with a subtag describing the particular strategy. The subtag *salience* was assigned when a participant reported choosing an object based on a feature which stood out. The subtag *preference* was assigned when the participant reported selecting an object because they preferred one of its attributes, e.g., “I like blue more than red" as a reason to pick a blue object. *preference* is similar to guessing but is more likely to result in consistent choices. Subtag *visual_resemblance* was assigned when the explanation involved picking an object based on it resembling the message in some way, e.g., picking a circle because the paint splodge in the message looks more like a circle. Other strategies were assigned the subtag *other*.

Like in Mayn & Demberg [14], explanations of some of our participants revealed that they had misunderstood the task. They understood the fact that not all messages were available to the sender to mean that not all objects could be referred to. For instance, in the top panel of Fig 1, someone who misunderstood the instructions that way might say that the speaker was not allowed to refer to the blue circle or the red square because the messages “blue" and “square" were not available. This way of thinking would lead participants to always respond incorrectly to items in the simple condition because the target contains an inexpressible feature. Such explanations were labeled *misunderstood_instr*. Since understanding the instructions this way essentially means that participants were solving a different task, we excluded participants whose explanation for either trial revealed that kind of reasoning from all analyses.

Additionally, some participants’ explanations revealed that they applied an inverse reasoning strategy where they interpreted the message non-literally. For the example in the top panel of Fig 1, someone applying such a strategy would select the blue circle since it is the only *non-red* object. Such responses were labeled *odd_one_out*. This kind of reasoning also reflects a fundamentally different strategy, which results in never selecting the target but does not reflect a lack of reasoning sophistication, unlike guessing. Therefore, we excluded participants whose explanation for either trial revealed that they were applying the *odd_one_out* strategy from all main analyses but we examine them more closely in the analysis of annotations.

Responses which were not clear or which did not reveal anything about the participant’s reasoning strategy were labeled *unclear*. Participants whose responses were labeled *unclear* were kept in the regression analysis.

### Reasoning types

The idea central to the work by F&D is that of reasoning types: that three Bayesian probabilistic models of different reasoning complexity can be defined which make different predictions about the reference game.

In the Rational Speech Act model [7] and closely related Iterated Best Response models [8], listener *L*_*N*_ and speaker *S*_*N*−1_ are Bayesian agents who recursively reason about each other and alternative possible utterances by the speaker and interpretations by the listener. Models of different recursion depth make different predictions for the performance on the reference game.

The simplest model is a *literal listener L*_0_ who interprets the speaker’s message *m* literally and assigns an equal probability to all objects *o* of which the message is literally true. The probability distribution of the literal listener is defined as $L_{0}(o|m)\propto [[m]]\cdot P(o)$, where [[*m*]] is the boolean function of the message’s meaning, corresponding to whether it is literally true of the object *o*, and *P(o)* is the prior probability that the object will be referred to. *P(o)* is often defined as the object’s salience. *L*_0_ will only be able to correctly solve unambiguous trials. On both simple and complex critical trials, it will assign equal probability to the target and the competitor since they both match the message.

The next model in terms of complexity is *L*_1_, which F&D termed *exhaustive listener* since its predictions closely align with an exhaustive interpretation in pragmatic inferences, which is a formalization of pragmatic inferencing from theoretical linguistics. *L*_1_ reasons about a literal speaker *S*_0_, who is equally likely select any true utterance to refer to an object. *L*_1_ is defined as $L_{1}(o|m,\alpha_{L_{1}})\propto exp(\alpha_{L_{1}}\;\cdot \;S_{0}(m|o;\alpha_{S_{0}}\to \infty )$, where $\alpha_{S_{0}}$ and $\alpha_{L_{1}}$ are the speaker’s and the listener’s temperature, or rationality, parameter respectively, which controls the degree to which the agent maximizes their utility. *L*_1_ is able to solve simple but not complex trials. Let’s show why that is the case. On simple trials, *S*_0_ has only one way of referring to the target, which is the uttered message, because the other feature of the target is inexpressible, and two ways of referring to the competitor. Therefore, *L*_1_ will reason that the message on simple trials is twice as likely to refer to the target, since *S*_0_ will use it 100% of the time to refer to the target and only 50% of the time to refer to the competitor (the other 50% of the time, *S*_0_ will use the other available message). On the complex condition, *L*_1_ will be at chance since both the target and the competitor have two messages which can be used to refer to them.

Finally, the most complex listener model is the classic pragmatic listener model *L*_2_. It is, in fact, more common in the literature to refer to the pragmatic listener model which reasons about *S*_1_ as *L*_1_ and not *L*_2_. However, we call it *L*_2_ here, following F&D to distinguish it from the *L*_1_ listener model reasoning about a literal speaker *S*_0_. *L*_2_ is a pragmatic listener model that is most commonly used in RSA modeling literature. *L*_2_ reasons about the pragmatic speaker *S*_1_, who, in turn, reasons about the literal listener *L*_0_. *S*_1_ is defined as $S_{1}(m|o,\alpha_{S_{1}})\propto exp(\alpha_{S_{1}}\cdot (\log L_{0}(o|m)-Cost(m))$. The speaker seeks to maximize their informativity, that is, the probability that the literal listener *L*_0_ will correctly identify the intended referent, while minimizing utterance cost. *L*_2_ is then defined as $L_{2}(o|m)\propto S_{1}(m|o;\alpha_{S_{1}\to \infty })\;\cdot \;P(o)$, where *P*(*o*), like in *L*_0_, is the prior probability of the object being referred to. It mirrors the definition of *L*_0_, with the speaker probability distribution instead of the literal meaning. *L*_2_ is powerful enough to solve both implicature types. It will solve the complex condition by reasoning that *S*_1_ will prefer to use the uttered message to refer to the target since the competitor has an unambiguous message (“triangle" in the example in the bottom panel of Fig 1) to refer to it.

F&D fit one of these models to each individual participant. They found that all three listener models were represented, with responding consistent to the predictions of *L*_1_ being the most common.

Notably, cognitive plausibility of *L*_1_ has been questioned: *L*_1_’s ability to solve the simple condition rests on the asymmetry in available messages and assumes a literal speaker. In an earlier study [17], we found that, when presented with a speaker who is explicitly literal (a simple computer program whose mechanism is explained to the participants), participants behaved not as *L*_1_ but as *L*_0_ and interpreted the messages literally, failing to translate the asymmetry in available messages into asymmetry in their interpretations. This suggests that *L*_1_ may not be an accurate description of the reasoning process if taken literally, but rather that it should be thought of a stable state of problem complexity, and that other, processing-level models may be needed to explain the reasoning of people who are able to solve the simple but not the complex condition. While proposing such models is beyond the scope of this contribution, we speculate about possible approaches in the discussion.

We would expect the following reported strategies to correspond to the three reasoning types: participants who belong to the *L*_0_ reasoning class should report a *guess* strategy in both the simple and the complex conditions; those in the *L*_1_ class should report *correct_reasoning* in the simple condition and *guess* in the complex condition; and those in the *L*_2_ class should report *correct_reasoning* in both conditions.

### Individual differences in pragmatic processing

We hypothesize that three individual difference traits may modulate people’s pragmatic responding on the reference game: working memory, Theory of Mind and logical reasoning. Here, we review related work which has investigated the effect of these individual differences for other pragmatic phenomena.

We provide a detailed justification for the selected cognitive constructs below, but we also would like to briefly discuss which cognitive traits we did not select as we do not expect them to be related to pragmatic reasoning in our task of interest. Pragmatic and discourse processing has been shown to be modulated by linguistic abilities, such as vocabulary knowledge [18] and exposure to print [19]. The reference game task we are investigating here is not language-based and uses symbols as possible messages and referents. Thus, for this particular setting, we did not expect linguistic skills to have an effect and therefore did not investigate them.

Some studies in pragmatics have observed effects of personality traits, such as openness [20], as well as of socio-pragmatic traits, as measured by the Autism Sprectrum Quotient (AQ) [4], and self-reported thinking dispositions, such as Need for Cognition (NfC) [21]. We do not include these measures in the current study for multiple reasons. First, in our early-stage pilot [22] we did not find any evidence of NfC and AQ in the reference game. Second, in the current study we are more interested in cognitive traits which modulate pragmatic processing. This is not to say that personality or neurodevelopmental factors do not play a role, but we leave their investigation to future work.

#### Working memory.

There is mixed evidence of the role of working memory capacity (WMC) in pragmatic inferencing. While it has been argued that deriving a nonliteral intepretation is effortful and may depend on sufficient cognitive resources, other studies have found no effect of working memory, suggesting a more automatic derivation process.

In the domain of scalar implicatures, De Neys et al. [23] found that their participants were more likely to accept under-informative statements when under cognitive load. Similarly, Antoniou et al. [24] found that participants with higher working memory capacity, as measured using the Backward Digit Span Test and the Reading Span task, were more likely to reject under-informative statements. Yang et al. [4] found that participants with higher WMC were better able to incorporate contextual information into scalar implicature derivation. Heyman & Schaeken [3], on the other hand, found that WMC, as measured by a modified Operation Span Task, was not a significant predictor for the rate of scalar implicature derivation for their participants.

Fairchild & Papafragou [5] investigated whether individual differences in executive function (EF), a construct closely related to working memory capacity, predicted pragmatic responding in scalar implicatures, as well as indirect request and metaphor comprehension. They found that EF was a significant predictor of pragmatic responding on its own, but when a measure of Theory of Mind was added to the model, EF ceased be significant, suggesting that EF may play an auxiliary role in that it supports perspective taking.

Ryzhova et al. [6] did not find an effect of verbal working memory, as measured by Reading Span, on the rate of derivation of inferences about event typicality when encountering a seemingly redundant mention of a highly typical event.

Finally, in an early-stage pilot study [22], we did not find an effect of WMC, as measured by Operation Span, on pragmatic responding in the reference game. However, there are several potential reasons why a true effect of WMC did not come out. First, most of the participants of the pilot had a mean score of 0.93 out of 1 on OSpan, meaning that most of their participants were at ceiling, which means that there may not have been sufficient variability for the effect to come out. Second, the effect may have been too small to be identified given the small sample size (*N* = 68). Third, in the pilot, we only used one measure for WMC, which may have made it harder to detect the effect if the measure is noisy and additionally measured factors unrelated to working memory.

#### Theory of Mind.

Theory of Mind (ToM), also sometimes called mentalizing [2] or mindreading, is the ability to attribute mental states, such as beliefs and desires, to oneself and others (see [25] on subtle distinctions between these terms). It may be instrumental in pragmatic inferencing, as inferencing may involve taking the speaker’s perspective into account and reasoning about why they chose the utterance they did.

A large number of studies investigated the relationship between Theory of Mind and pragmatic processing in children and adolescents and how it changes with age. Longitudinal studies on the role of Theory of Mind in metaphor comprehension have shown that ToM supports metaphor comprehension in children of ages 8-9 but reliance on ToM diminishes with age [26–28]. A positive relationship between ToM skills and comprehension of irony and deceit has been shown [29,30].

Studies in clinical populations, such as adults with schizophrenia (see [31] for a review) have observed a co-occurrence of ToM impairment with impairment of pragmatic skills. There is also a debate in the literature about ToM impairment, as well as its role in pragmatic processing in adults with autism, with some studies claiming that there is a ToM impairment in individuals with autism [32,33], and other studies calling for caution due to the fact that many existing ToM tests are based on neurotypical conception of social norms and behavioral expressions and may unfairly disadvantage neurodivergent populations [34,35].

A growing body of work suggests a link between Theory of Mind and pragmatic processing in neurotypical adults as well, which is our population of interest. Several studies observed that ToM facilitated processing of indirect requests. Marocchini et al. [36] showed that participants with higher ToM, as measured by the Strange Stories task [37], showed facilitated processing of conventionalized indirect requests in a self-paced reading paradigm. Trott & Bergen [2] and Fairchild & Papafragou [5] also observed that ToM, measured by the Short Story Task [38] and a composite score of the Strange Stories task and the Reading the Mind in the Eyes Test [39] respectively, modulated indirect request comprehension. Higher Theory of Mind abilities has also been found to be predictive of humor comprehension: Bischetti et al. [40] showed that older adults with higher ToM demonstrated better comprehension of mental but not phonological jokes, and Loy & Demberg [41] showed that participants with better mentalizing abilities were better at tracking whether a speaker was likely to produce a humorous utterance. ToM abilities have also been shown to modulate scalar implicature derivation and metaphor comprehension in neurotypical adults [5].

#### Logical reasoning.

Finally, we hypothesized that the ability to reason abstractly and logically may play a role in deriving a pragmatic interpretation in the reference game. Evidence for the role of logical reasoning ability in pragmatic inferencing is limited and somewhat mixed. Heyman & Schaeken [3] found that scores on the Cognitive Reflection Test (CRT) [42], which is assumed to measure reflectivity and the ability to override an intuitive response, were not predictive of their participants’ rate of scalar implicature derivation.

On the other hand, Ryzhova et al. [6] found an effect of logical reasoning, as measured by a composite score of CRT and Raven’s Progressive Matrices (RPM) [43], on the rate of derivation of atypicality inferences: participants with higher scores were more likely to derive a habituality inference following a mention of a highly typical event.

In our early-stage pilot [22], we investigated the effect of the reasoning-related measures used in the above studies, Raven’s Progressive Matrices and the Cognitive Reflection Test, on reference game performance. We observed an effect of Cognitive Reflection in both pilot experiments, whereas an effect of the RPM score only emerged in one. We therefore hypothesize that these two measures have a positive effect on pragmatic responding in the reference game, which is likely to come out more robustly with a larger sample size.

The extent to which CRT and RPM tap into the same versus distinct constructs has been debated in the literature [44,45]. Some studies argue that CRT taps into the tendency towards miserly processing [46] and the ability to inhibit the intuitive response [48], whereas other studies suggest that it may be primarily a cognitive measure strongly associated with intelligence [49,50]. Stanovich [45]’s extended conceptual model of Type 1 and Type 2 processing suggests that CRT may reflect individual differences in rational thinking dispositions or cognitive styles (or *reflective mind*), whereas RPM reflects individual differences in fluid intelligence (or *algorithmic mind*), both of which are part of intentional, slower Type 2 processing. Therefore, CRT may reflect higher-level cognitive control needed to start a cognitive operation which the algorithmic mind then carries out.

Given the results of the Principal Component Analysis, in our analysis we mostly treat the two measures as reflecting the same ability, which we call *logical reasoning*, but in the discussion we return to this distinction and additionally examine the effects of these two individual difference measures separately.

## Experiment

This study was conducted with the approval of the Ethical Review Board (ERB) of the Department of Computer Science at Saarland University (Approval No. 20-10-2). All participants gave written consent according to the policies set forth by the ERB.

### Participants

300 self-reported native English speakers with an approval rate of 95% or higher were recruited via the crowdsourcing platform Prolific. Recruitment took place on December 6 and 7, 2022, and the first study session was conducted on the same days due to the efficiency of the platform. In order to ensure variability in the sample, participants were recruited from 3 streams: 100 participants who have a university degree, 100 participants who are in the process of obtaining a technical or community college or a university degree, and 100 participants who completed high school or did not have formal qualifications. 222 participants returned to participate in the second session.

### Procedure

The experiment was conducted in two sessions. In the first session, which took place on December 6 and 7, 2022, participants completed the reference game, Backward Digit Span, Cognitive Reflection Test, and Raven’s Progressive Matrices, in that order. The first session took participants about 25 minutes to complete, for which participants were compensated £3.16. Only participants who did not meet the exclusion criteria for any of the tests from the first session were reinvited for the second session, which took place one week later, between December 12 and 15, 2022. In the second session, participants completed the OSpan task, the Short Story Task, and the Reading the Mind in the Eyes task, in that order. The second session took participants about 45 minutes to complete, for which participants were compensated £6.44.

**Individual difference tests.**

**Operation Span (OSpan).** As one of the measures of working memory capacity, we used the automated online version of operation span (OSpan) based on Unsworth et al. [47]. Participants were asked to verify the correctness of a total of 75 basic math equations, presented in sets of 3 to 7, while holding letters in memory, which appeared one by one at the end of each equation. Participants were instructed to not write anything down and to not use any aids for solving the equations or memorizing the letters. The optimal set length and equation difficulty was determined by conducting a set of pilot studies. This was done to avoid a ceiling effect. First, participants saw the left hand-side of the equation, e.g., (4+3)×2=?. Then, once they had solved it, they clicked a button to proceed to the next screen, on which a number was presented, and participants had to judge whether the number is the correct solution of the equation or not by clicking on one of two buttons. After that, a letter appeared on the screen for 800 ms, after which either the next equation was shown or, if it was the end of the set, the participant was prompted to type in all the letters. To ensure that participants did not take too long on the equations, a brief training phase in the beginning was used to estimate the time a participant needed to solve the equations. The maximum allowed time was set to the mean + 2.5 standard deviations of the time a participant took during the training phase. If a participant did not verify an equation during that time, a timeout message appeared for 800 ms, after which the letter was shown. After each set, participants were asked to type in the letters in the order in which they had appeared. The score was computed using the Partial Credit Unit Score (PCU) method from Conway et al. [51] as the mean proportion of letters within each set which were recalled correctly (i.e., the correct letter was recalled in the correct position). Participants whose accuracy on the equations was below 80% were excluded from analysis.

OSpan has demonstrated strong psychometric properties. Internal consistency, measured by Cronbach’s *α*, typically ranges fron .7 to .8 [47,51,52], while test-retest reliability over multiple weeks has found to be within the .7-.83 range [47,51]. Construct validity is supported by positive correlations with other working memory measures, such as Reading Span and Counting Span [52], as well as with fluid intelligence tasks like Raven’s Progressive Matrices [47]. These findings indicate that OSpan is a reliable measure of working memory capacity.

#### Backward Digit Span (BDS)

[53] was included as as the other measure of working memory capacity. Participants were presented with digit sequences of increasing length, from 2 to 8. There were 2 trials for each sequence length. Digits were presented one by one for 1000 ms. At the end of each set, participants were asked to type in the digit sequence in reverse. Participants were instructed not to write down the digits and not to use any aids to help them remember. Copying, pasting, or adding characters anywhere except the end of the string was disabled in order to ensure that participants were retrieving the digit string from memory and performing the reversal in their heads.

If the participant got both sequences of the same length wrong, the experiment ended. The score is the highest sequence length for which a participant got both trials right. Sequence length ranged from 2 to 8. Participants who did not recall both trials correctly for any length were excluded from analysis.

BDS has been shown to have adequate psychometric properties. Internal consistency, measured by Chronbach’s *α*, ranges from .59 to .93 [54,55], and test-retest reliability in the range between .64 and .83 has been reported [54–56]. De Paula et al. [57] reported moderate split-half reliability of .598. Construct validity of BDS is supported by positive correlations with other memory span measures [54], as well as with fluent intelligence [55].

While OSpan is considered a complex span task, since it involves performing a secondary task while keeping information in memory, BDS is typically considered a simple span task. Simple span tasks are thought to primarily engage short-term memory and emphasize maintenance rather than processing, whereas complex span tasks like OSpan involve both storage and processing components [58,59]. However, it has been argued that BDS can be considered a measure of working memory as opposed to only short term memory, as it requires the manipulation of information in memory and not just storage, unlike the forward digit span [55,58,60,61]. Despite these distinctions, simple and complex span tasks have been consistently shown to be closely related. Meta-analyses suggest that both types of tasks reflect the same underlying cognitive mechanisms – rehearsal, storage and updating – but to different degrees, with simple spans placing greater emphasis on storage [62,63]. The Principal Component Analysis conducted in this study supports this view, as both OSpan and BSD loaded strongly onto the same principal component.

#### Raven’s Progressive Matrices (RPM)

[43] was included as a test of logical reasoning ability. On each trial, participants were presented with an incomplete pattern and 6-8 options out of which they needed to select the missing piece to complete the pattern.

We used a shortened 10-question version of the full Raven’s Standard Progressive Matrices Test (RSPM), since a shortened 9-question version of the test has been found to correlate almost perfectly with a full-length RSPM test [64]. The 10-question version consisted of the 9-question Form A test from Bilker et al. [64] and the most difficult item from the Advanced Progressive Matrices Test (RAPM) (item 36), which was added as the final question to account for possible ceiling effects, following Scholman et al. [65].

The questions were presented in ascending order of difficulty. While participants were solving the task, a timer remained on the top of the screen, which showed the total amount of time participants had spent on the task up until that point. Participants were told that while their time isn’t limited, they should avoid thinking too long. The score was computed as the number of correctly solved items.

In the preregistration, we stated that we would exclude participants who had taken an abstract reasoning test of this type in the last 6 months. However, due to experimenter error we did not ask participants that question. However, we collected participants responses about whether they recalled seeing any of the questions before. 20 out of 300 participants responded “Yes" or “Not sure", 8 of whom did not meet exclusion criteria for other tasks. The results section of the paper includes those 8 participants. Excluding these participants does not substantially change the posterior estimates of the effects or their credible intervals. Regression results with those participants excluded are reported in S1 Appendix.

The 9-item version of RSPM, which is identical to the version we use with the exception of the last item, has been shown to have good reliability (Cronbach’s *α* = .80, compared to *α* = .96 for the full 60-questions RSPM) [64].

#### Cognitive Reflection Test (CRT)

[42] was used as the other measure of logical reasoning ability. CRT has been argued to measure reflexivity, that is, how likely someone is to override the first intuitive response and engage in deeper reasoning. It has been shown to correlate robustly with measures of intelligence and in addition to that be a unique predictor in heuristics and biases tasks [44].

In our early-stage pilot study [22], we found a correlation of *r* = 0.45 between RPM and CRT, and Ryzhova et al. [6] found that their participants’ scores on RPM and CRT loaded onto the same principal components. Therefore we hypothesize that CRT also taps into logical reasoning ability and will load onto a principal component corresponding to logical reasoning in our study as well.

We used a 10-question version of CRT, with 6 critical questions, 3 verbal and 3 computational, and 4 decoy questions, presented in random order. This version was developed by Scholman et al. [65] and consists of questions from earlier versions of CRT [46,66–69]. The critical questions were “trick questions", where the intuitive answer does not match the correct answer. An example of a question is “If you were running a race, and you passed the person in 2nd place, what place would you be in now?". The intuitive answer is 1st place but the correct answer is 2nd place.

After each question, participants were asked whether they had seen it before. The score was computed as the proportion of correctly answered previously unseen critical questions. Since CRT has been shown to be affected by familiarity [69,70], participants who reported having seen 3 or more critical questions were excluded from analysis.

CRT has adequate psychometric properties. Moderate to good internal consistency has been reported by previous studies (Cronbach’s *α* ranging from .51 to .88 for different versions [39,46,67–69]). Construct validity has been supported by positive correlations with other measures of rational thinking, such as the belief bias task, temporal discounting, denominator neglect and conditional reasoning, as well as with Need for Cognition [46,67–69].

#### Short Story Task (SST)

[38] was used as a measure of Theory of Mind. It has been found to predict comprehension of indirect requests [2] and reading time of texts containing irony [71].

In the task, participants read the short story “The End of Something" by E. Hemingway. After reading the story, participants were asked if they had read the story before, and if so, how well they remembered it and whether they had read it in school or for pleasure. Then participants were asked to briefly summarize the story, after which they answered 8 open-ended questions about characters’ mental states along with 5 comprehension questions. Participants typed their answer to each of the comprehension questions in a textbox. An example of a mental state question is “Why does Marjorie take the boat and leave and what is she feeling at that moment?".

The response to each question was scored 0-2 using the rubric from Dodell-Feder et al. [38]. The total score was the sum of the scores for the individual mental state inference questions. Thus, the possible range of scores was 0-16. Participants who scored less than half (i.e., fewer than 5 out of 10 points) on the comprehension questions were excluded from analysis.

Given the broad range of content covered by individual items in the SST, its internal consistency is moderate, with Cronbach’s *α* ranging from .27 to .51 for comprehension questions and from .54 to .63 for mental state reasoning questions [38,72,73]. Construct validity has been supported by positive correlations with the Reading the Mind in the Eyes Test (RMET) and the fantasy subscale of the Interpersonal Reactivity Index (IRI) [72,74]. Additionally, positive associations have been reported with both verbal and non-verbal intelligence by Dodell-Feder et al. [38] and Giordano et al. [72], although these findings were not replicated by Jarvers et al. [73].

#### Reading the Mind in the Eyes Test (RMET)

[39] was used as the second measure of Theory of Mind. It has been suggested that RMET may tap more into emotion recognition than into mentalizing [75]. It has also been observed that RMET is strongly related to vocabulary knowledge and moderately related to emotion perception and self-reported cognitive empathy [76], suggesting that RMET may be reflective of those individual differences, possibly to a larger extent than of mentalizing [77].

Despite these limitations, we opted for using it as a ToM measure for several reasons. It is the most commonly used test for Theory of Mind, and unlike most ToM tests, it does not show ceiling effects for neurotypical adults [78]. Also, it has been shown to correlate with other Theory of Mind measures, including our other measure, the Short Story Task (Dodell-Feder et al. [38] report a correlation of *r* = .49, 95% CI = [.28, .68]).

In this task, participants are presented with 36 pictures of people’s eyes, presented one-by-one, and are asked to select the emotion which the person is experiencing from the 4 available options. The score is the number of correctly answered questions.

According to a review of 1,461 studies [79], the psychometric properties of the RMET are reported to be mixed but generally acceptable. Internal consistency, measured by Cronbach’s *α* across 132 studies, ranged from .17 to .96, with 57.9% of estimates falling below .70. McDonald’s *ω*, reported in 18 studies, ranged from .43 to .85 (mean = .65), with 61.1% of values below .70. Split-half reliability, assessed in 8 studies, ranged from .50 to .75, with 55.6% of estimates below .70. The validity of the RMET is commonly supported by its ability to discriminate between neurotypical adults and those with autism (151 studies), schizophrenia (29 studies), and between males and females (19 studies). Convergent validity has been assessed using other ToM measures, as well as measures of empathy and emotion recognition. Discriminant validity has been demonstrated by the RMET’s lack of correlation with fluid intelligence, verbal ability (though see [76]), and executive functioning [79].

### Results

We first examine the results of the reference game to verify that the effect of condition (simple and complex implicature) observed by F&D and Mayn & Demberg [14] holds for our sample and to confirm that there is individual variability.

Next, we conduct an analysis where we add the individual differences we collected as predictors to the regression model to examine whether they explain performance on the reference game.

#### Participant exclusion.

Of the 300 participants who participated in the first session, 8 were excluded for accuracy below 80% on the unambiguous trials on the reference game. Further 27 participants were excluded because they were familiar with 3 or more of the 6 critical questions on the Cognitive Reflection Task. Further 8 participants were excluded because they did not correctly recall both sequences for any length in Backward Digit Span.

The remaining 257 out of 300 participants were reinvited to participate in the second session, and 222 of them did. Of those 222, 194 participants passed exclusion criteria for all of the tasks.

Thus, 292 participants had passed the exclusion criterion for the main task, 194 of whom also passed exclusion criteria for all individual difference tasks.

Annotations of participants’ explanations revealed that a total of 25 participants (23 of whom were part of the 292 retained for the main task, and 15 of whom were part of the 194 retained for the individual differences analysis) misunderstood the instructions of the main task, and 15 participants (all of whom were part of the 292 retained for the main task, and 12 of whom were part of the 194 retained for the individual differences analysis) applied the odd-one-out (inverse reasoning) strategy not captured by any of the RSA models. Those people were also excluded from analysis.

As a result, 254 participants (149 female, 105 male; mean age = 35.6 years (SD = 13.6, 3 participants did not report their age)) entered the replication analysis without individual differences, and 167 participants (97 female, 70 male; mean age = 36.3 years (SD = 13.6, 2 participants did not report their age)) entered the main analysis with individual differences.

#### Replication of effects from F&D (2016).

First, we examine the performance on the reference game task to make sure that we replicate the effect of condition found in F&D, as well in Experiment 4 of Mayn & Demberg [14], which used the same stimuli as we use here: both studies found better performance on simple trials compared to complex trials and better performance on complex trials compared to the chance baseline.

**Fig 2:**
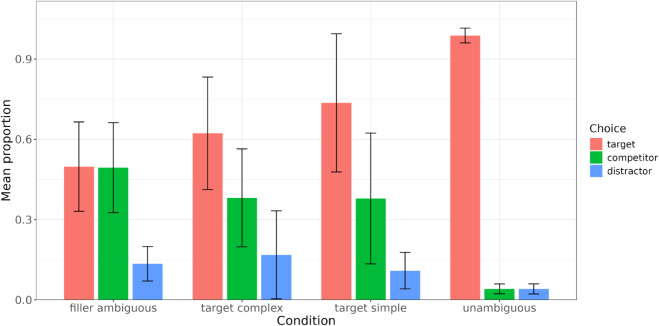
Average proportions of responses in the reference game per utterance type. Error bars represent standard deviations.

Fig 2 shows average proportions of choices (target, competitor and distractor) for each trial type. Since the proportions are averaged across participants, they may not necessarily sum to one for each trial type. As expected, performance on ambiguous trials is at chance (mean proportion of target responses = 0.50, *SD* = 0.17) since there are two identical objects and which one of them is target is determined via a coin flip. Performance on unambiguous trials is at ceiling (mean proportion of target responses = 0.99, *SD* = 0.03), which indicates that participants understood the task. Finally, performance on the critical simple condition (mean proportion of target choices = 0.72 (*SD* = 0.27)) appears to be better than on the complex condition (mean proportion of target choices = 0.62 (*SD* = 0.21)). We will confirm that the difference between the two conditions is significant in a regression analysis.

These results are consistent with the original study by F&D, whose results, however, showed a somewhat numerically stronger difference between the simple and the complex conditions (mean proportion of target choices in the simple condition = 0.79 (*SD* = 0.19), in the complex condition = 0.57 (*SD* = 0.18)). These averages were computed by us using F&D’s data. In their paper, F&D report pooled proportions of choices (77% target choices in the simple condition and 57% in the complex condition; Fig 2 in F&D). However, we opted for reporting average proportions here instead as they provide a clearer view of the data’s spread.

F&D observe somewhat higher performance on the simple condition and lower performance on the complex condition than the current study. That is not very surprising given Mayn & Demberg [14]’s findings that performance on the simple condition is inflated for the original stimuli because of reasons related to the stimuli and unrelated to pragmatic reasoning.

**Fig 3:**
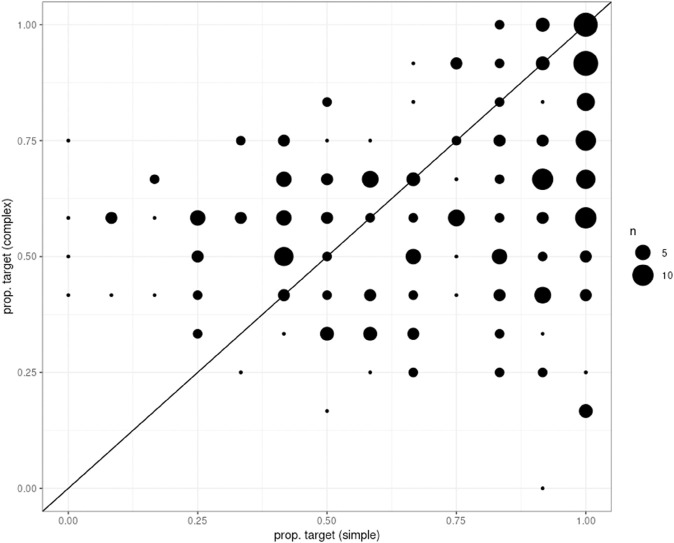
Proportion of target choices on simple and complex trials by participant. Dot size indicates number of participants.

Interestingly, we observe more individual variability in our sample than F&D and Mayn & Demberg [14]. Fig 3 shows proportion of target selection in the two critical conditions by participant. We find that the effect is quite graded and, interestingly, that quite a few participants appear to perform somewhat better on the complex condition than on the simple condition, corresponding to the points above the diagonal. That is something that none of the RSA models predict. We will look at those individuals more closely in our analysis of annotations. However, consistent with the RSA’s predictions, there are many more participants on or below the diagonal than there are above it (70% vs. 30%); and, of course, some variability can be attributed to guessing.

Next, in order to verify the difference in performance on the simple and complex conditions, we conduct Bayesian logistic regression using the *brms* package in R. Following F&D, we exclude unambiguous trials, as well as trials on which participants chose the distractor (which amounts to 1.2% of trials), from this analysis. Trial correctness, a binary variable, is regressed onto condition (helmert-coded, simple vs. rest and complex vs. ambiguous), trial number (mean-centered), participant age (mean-centered), target position (left, middle or right, dummy-coded with left as the reference level), message type (sum-coded, -1 = shape, 1 = color), and the interaction of trial number with condition. The random effect structure included per-participant and per-item random intercepts and per-participant random slopes for condition, message type, and trial number.

For all effects, very wide weakly informative priors centered at 0 were set. The full model equation and a discussion of the priors is included in S2 Appendix. We ran four chains of 6000 iterations each, with the first 1000 iterations of each chain discarded as warm-up.

To assess the evidence for the effects, we examined whether the credible intervals for the parameter estimates included zero. If the credible interval did not include zero, we concluded that there was a meaningful relationship between the predictor and the dependent variable. The regression results are reported in Table 2.

**Table 2:** Regression model output for the model without individual differences (*N* = 254) and for the model with individual differenceseak (*N* = 167). Effects for which the 95% CrI does not include 0 are bolded.

Effect	Model without IDs (*N* = 254)		Model with IDs (*N* = 167)	
	Estimate	95% CrI	Estimate	95% CrI
Intercept	**0.63**	[0.49, 0.77]	**0.46**	[0.23, 0.68]
condition1 (simple vs. rest)	**1.30**	[1.03, 1.58]	**0.97**	[0.60, 1.34]
condition2 (complex vs. ambig)	**0.62**	[0.44, 0.80]	–	–
trial number	**0.00**	[0.00, 0.01]	**0.01**	[0.01, 0.02]
participant age	-0.00	[-0.01, 0.00]	**-0.02**	[-0.03, -0.01]
msgtype (color vs. shape)	**-0.08**	[-0.14, -0.01]	-0.10	[-0.23, 0.02]
targetpos (middle vs. left)	**0.36**	[0.24, 0.49]	**0.53**	[0.33, 0.74]
targetpos (right vs. left)	**-0.20**	[-0.33, -0.08]	**-0.21**	[-0.40, -0.03]
condition1 : trial	-0.01	[-0.01, 0.00]	-0.01	[-0.02, 0.00]
condition2 : trial	0.01	[-0.00, 0.01]	–	–
logical reasoning	–	–	**0.22**	[0.06, 0.38]
memory	–	–	0.06	[-0.10, 0.22]
ToM	–	–	**0.26**	[0.10, 0.42]
condition1 : logical reasoning	–	–	**0.45**	[0.13, 0.78]
condition1 : memory	–	–	0.24	[-0.07, 0.56]
condition1 : ToM	–	–	0.16	[-0.16, 0.48]

The model confirms that participants’ performance on the simple condition was better than on the complex condition ($\hat{\beta }$ = 1.30, 95% CrI = [1.03, 1.58]), and performance in the complex condition was above chance ($\hat{\beta }$ = 0.62, 95% CrI = [0.44, 0.80]). There was a very small positive effect of trial number ($\hat{\beta }$ = 0.00, 95% CrI = [0.00, 0.01]). Since the effect size is so small, we believe that we can still say that the assumption that participants retain one reasoning complexity type throughout the experiment holds. Participants were less likely to interpret the message correctly if the message was a color as opposed to a shape ($\hat{\beta }$ = -0.08, 95% CrI = [-0.14, -0.01]), and they were more likely to select the target if it was in the middle as opposed to on the left ($\hat{\beta }$ = 0.36, 95% CrI = [0.24, 0.49]), and on the left compared to the right ($\hat{\beta }$ = -0.20, 95% CrI = [-0.33, -0.08]).

Overall, we see that we very closely replicate the original study by F&D, and that the effect sizes for condition are very close to the those reported in the original study (see Table 3 for model results of F&D).

**Table 3:** Regression model output from Franke & Degen (2016) (F&D; *N* = 51) and from Experiment 4 of Mayn & Demberg (2023) (M&D; *N* = 60). Effects for which *p<*0.05 for F&D and effects for which the 95% CrI does not include 0 for M&D are bolded.

Effect	F&D (2016) (*N* = 51)		M&D (2023) (*N* = 60)	
	Estimate	SE; *p*	Estimate	95% CrI
Intercept	-0.15	0.11; 0.18	0.20	[-0.20, 0.61]
condition1 (simple vs. rest)	**1.28**	0.12; < 0.0001	**1.24**	[0.67, 1.86]
condition2 (complex vs. ambig)	**0.44**	0.13; < 0.001	**0.42**	[0.03, 0.85]
trial number	0.00	0.00; < 0.3	**0.01**	[0.00, 0.02]
msgtype (color vs. shape)	-0.02	0.12; < 0.85	0.22	[0.03, 0.85]
targetpos (middle vs. left)	**1.28**	0.14; < 0.0001	**0.38**	[0.11, 0.64]
targetpos (right vs. left)	**0.74**	0.13; < 0.0001	-0.04	[-0.31, 0.23]
condition1 : trial	0.00	0.01; < 0.9	**-0.02**	[-0.03, -0.00]
condition2 : trial	0.01	0.01; < 0.9	**0.02**	[0.00, 0.03]

#### Analysis of annotations.

Next, we inspect the strategies reported by participants on the main task and how those strategies related to participants’ performance.

First, we examine the relationship between their reported strategy and their tendency to select the target (i.e., pragmatic responding). For this analysis, we also include the strategies *misunderstood_instr* and *odd_one_out*, which we excluded for the regression analysis. Thus, this analysis only excludes the 8 participants whose accuracy on the unambiguous condition was below 80%, with *N* = 292 participants remaining.

**Fig 4:**
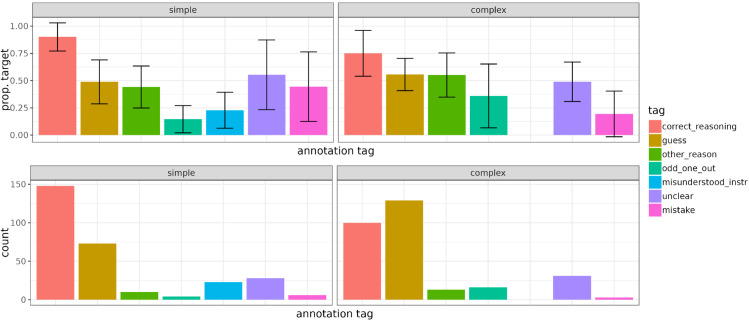
Mean proportion of target selections per annotation tag and the frequency of that tag in the two critical conditions. Error bars represent standard deviations.

Fig 4 shows the mean proportions of target selections (averaged across participants) and the frequency of each annotation tag. We can see that the two annotation tags that were by far most common in both conditions are *correct_reasoning* and *guess*. In the simple condition, there is a higher proportion of *correct_reasoning* responses than in the complex condition (50.7% (148 out of 292) vs. 34.2% (100 out of 292)) and a lower proportion of *guess* responses (25% (73 out of 292) vs. 44.2% (129 out of 292). We also see that the mean proportion of target selections corresponding to the *correct_reasoning* tag is higher in the simple condition than in the complex condition (0.90 (*SD* = 0.13) vs. 0.75 (*SD* = 0.21)), suggesting that even when people reported a correct strategy in the complex condition, at the population level, they applied it less consistently than in the simple condition. We see that the strategy labeled *odd_one_out* corresponds to a relatively low proportion of target responses: 0.15 (*SD* = 0.13) in the simple condition and 0.36 (*SD* = 0.29) in the complex condition. That is because *odd_one_out* strategy picks out the object which is unlike the other two, which is most often the distractor. *misunderstood_instr* only appears in the simple condition, since it involves misunderstanding the meaning of unavailable messages, and in the complex condition, messages for all features of the three objects are available. *misunderstood_instr* has an average proportion of target responses of 0.23 (*SD* = 0.17), meaning that people who misunderstand the instructions might occasionally select the target but most of the time select the competitor or the distractor. *other_reason* responses – salience, preference and other strategies fall into that category – are very infrequent and correspond to about half of target selections (0.44 (*SD* = 0.19) in the simple condition and 0.55 (*SD* = 0.20) in the complex condition), which is close to guessing.

Overall, at least at the population level, reported strategies appear to match average proportion of target selections, with a high proportion of target selections for *correct_reasoning*, target proportion of near 50% for *guess* and a low proportion of target selections for *misunderstood_instr* and *odd_one_out*. We will examine the relationship between individual participants’ performance and their reported strategies in more detail in the next section.

#### Grouping participants using LPA and reported strategies.

As can be seen in Fig 3, there is a lot of individual variability in our data and participants’ performance appears to be gradient as opposed to building three clear clusters corresponding to the three reasoning types (*L*_0_, *L*_1_ or *L*_2_). However, since the three reasoning types serve as the theoretical explanation of the variation we observe, we start by conducting a clustering analysis where we examine whether such clusters can be identified and whether they have distinct profiles with respect to the individual differences.

We assessed the clustering tendency of the reference game data using the Hopkins statistic [80], which is an established measure for distinguishing data with meaningful cluster structure from randomly distributed data [81,82]. Values close to 0.5 indicate randomness, whereas values close to 1 indicate a cluster structure. Since the Hopkins statistic is stochastic, we computed it 10 times with different random seeds. The mean value was 0.93 (*SD* = 0.05), indicating a strong clustering tendency. This suggests that conducting a clustering analysis on this data is appropriate.

We perform latent profile analysis using the *tidyLPA* package in R on participants’ average performance on the simple and the complex conditions in order to identify clusters of participants in our data for the main task. LPA identifies clusters while remaining agnostic to the origins of those clusters. A 3-class model obtained a better fit to the data (AIC = -108.69, BIC = -73.31) than models with 1 (AIC = 1.55, BIC = 15.70) or 2 (AIC = -41.53, BIC = -16.77) classes. A 4-class model obtained a better fit than the 3-class model according to AIC but not according to BIC (AIC = -112.60, BIC = -66.61). Since we have a theoretical motivation to posit 3 groups of participants corresponding to the three RSA models, we examine the model with 3 classes.

**Fig 5:**
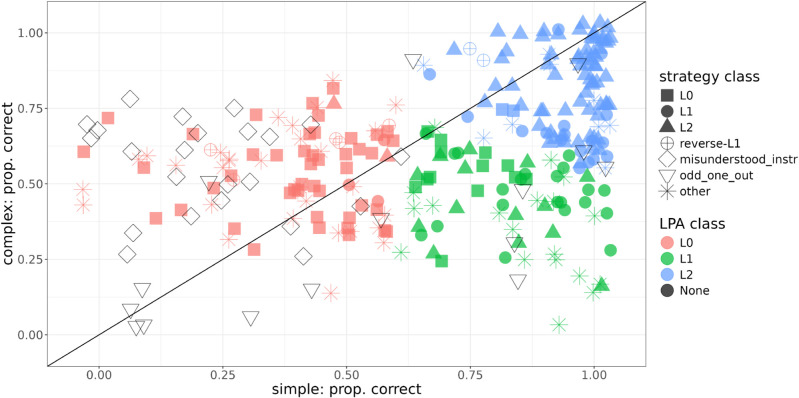
LPA classes vs. classes based on annotations.

Fig 5 shows the obtained classes. They correspond approximately to the predictions of the 3 idealized reasoning types. Participants whose performance on the simple condition is 0.6 or below are clustered together, roughly corresponding to the predictions of the *L*_0_ model. Another cluster includes participants with scores above 0.6 on the simple condition and below about 0.7 on the complex, corresponding to being able to solve simple but not complex trials, as the *L*_1_ model predicts. Finally, the third cluster represents participants with high performance on both conditions, corresponding to the predictions of the pragmatic listener model *L*_2_.

How do the classes identified by LPA based on performance relate to strategies that participants reported? In addition to the class assigned based on performance, Fig 5 also shows classes based on strategy. If the participant reported guessing on both conditions, we assign them to the *L*_0_ class based on their reported strategy. If their strategy was *correct_reasoning* in the simple condition and *guess* on the complex, they were assigned to the *L*_1_ class, whereas if their strategy was labeled *correct_reasoning* in both conditions, they were labeled *L*_2_. Note that participants assigned to the *L*_1_-class based on annotations perform counterfactual reasoning about *the speaker’s intent* (“if they had wanted to communicate X, they would have chosen the message Y"), but they only do so in the simple condition. We do not observe explanations referring to the distribution of messages available to a literal speaker, as the literal interpretation of the *L*_1_ RSA model would suggest. This supports our view that the *L*_1_ model should be viewed as a stable outcome capturing the difference in complexity between the two reference game conditions – only the simpler one of which can be solved correctly by participants belonging to this class – and not as an accurate processing-level account of people’s actual reasoning process.

Additionally, there were a few people who reported guessing in the simple condition but reasoning correctly on the complex – something that no RSA model predicts given that any model which is able to solve the complex condition should also be able to solve the simple condition – those people are assigned to the *reverse-L*_1_ class. Participants who employed a strategy other than *guess* and *correct_reasoning* on one or both conditions were assigned to the *other* class. Those strategies were: other reason – salience (5 in the simple condition, 3 in the complex condition), other reason – preference (4, 5), other reason – multiturn (2, 5), other reason – visual resemblance (0, 2), other reason – other (1, 2), unclear (32, 34), and mistake/changed_mind (7, 3).

Additionally, we also visualize participants who misunderstood the instructions or applied the *odd_one_out* strategy. Those participants were not used for the regression analysis or for the LPA clustering.

First, we examine the relationship between classes assigned by LPA based on performance and those assigned based on reported strategies. Table 4 is a confusion matrix which compares the two sets of classes. Overall, there is a decent degree of consistency between the two sets of classes. 45 out of 55 participants (81.8%) (who could be classified based on annotations, i.e., not counting participants in the *other* annotation class) in the *L*_0_ class based on LPA reported guessing as their strategy on both conditions. Only 21 out of 48 (43.75%) of participants who were assigned to the *L*_1_ class based on LPA reported correct counterfactual reasoning in the simple condition and guessing on the complex condition. 13 participants (27.08%) reported guessing on both conditions, so their relatively high performance on the simple condition is presumably caused by lucky guessing, and 14 participants (29.17%) reported performing correct counterfactual reasoning on both conditions, despite their relatively low performance on the complex condition. There are two possible explanations for this misalignment. Recall that participants were asked to reported their strategies at the very end, first for a simple trial and then for a complex trial. It could be that those participants figured out how to solve the complex condition during the task, resulting in lower performance on complex than on simple. It could also be that being asked to reflect on how they solved the simple condition made participants more likely to apply this reasoning to the complex condition on the final trial. Finally, 70 out of 89 participants assigned to the *L*_2_ class based on LPA (78.65%) reported applying correct reasoning in both conditions. 17 (19%) participants assigned to the *L*_2_ class reported guessing on the complex condition, corresponding to the *L*_1_ model, i.e., they were likely lucky in the complex condition.

**Table 4:** Confusion matrix comparing classes based on LPA (rows) and based on annotation (columns).

LPA Classes	Annotation Classes					
	L0	L1	L2	Reverse-L1	Other	Total
L0	**45**	3	2	5	31	86
L1	13	**21**	14	0	19	67
L2	2	17	**70**	2	10	101
Total	60	41	86	7	60	254

Interestingly, the *L*_1_ class is the least populous, both based on LPA (67 out of 254 participants, 26.38%) and based on reported strategies (41 out of 254, 16%) and also has the lowest consistency between classes based on performance and based on annotations. This contrasts with the findings of F&D, for whom *L*_1_ was the largest class among their participants. One possible reason for that might be sampling chance since F&D had a much smaller sample size (51 participants, as opposed to 254 in the current study). Another reason may be related to the difference in stimuli. Mayn & Demberg [14] showed that the original stimuli used by F&D, led to inflated performance in the simple condition. Thus, it may be that some people whose reasoning is actually more accurately described by the *L*_0_ model were classified into the *L*_1_ class. Some support for that hypothesis comes from comparing clusters obtained in Experiment 1 and Experiment 4 of Mayn & Demberg, which use F&D’s original stimuli and the geometric stimuli used in the current study respectively. In Experiment 1, there is a smaller *L*_0_ class and a larger *L*_1_ class, whereas in Experiment 4, it is the other way around.

Also, it is notable that there are quite a few people above the diagonal line, corresponding to higher performance on the complex condition than on the simple one, a pattern not predicted by the RSA. In particular, quite a few participants have a low proportion of target selections (below 0.3) on the simple condition. A large portion of those participants misunderstood the instructions, taking the fact that the target in the simple condition had an inexpressible feature to mean that it could not be referred to. There were presumably some unlucky guessers as well. Finally, there might have been participants who initially misunderstood the instructions but in the course of the experiment converged on a guessing strategy, thus reporting guessing at the end.

#### Individual difference measures.

**Fig 6:**
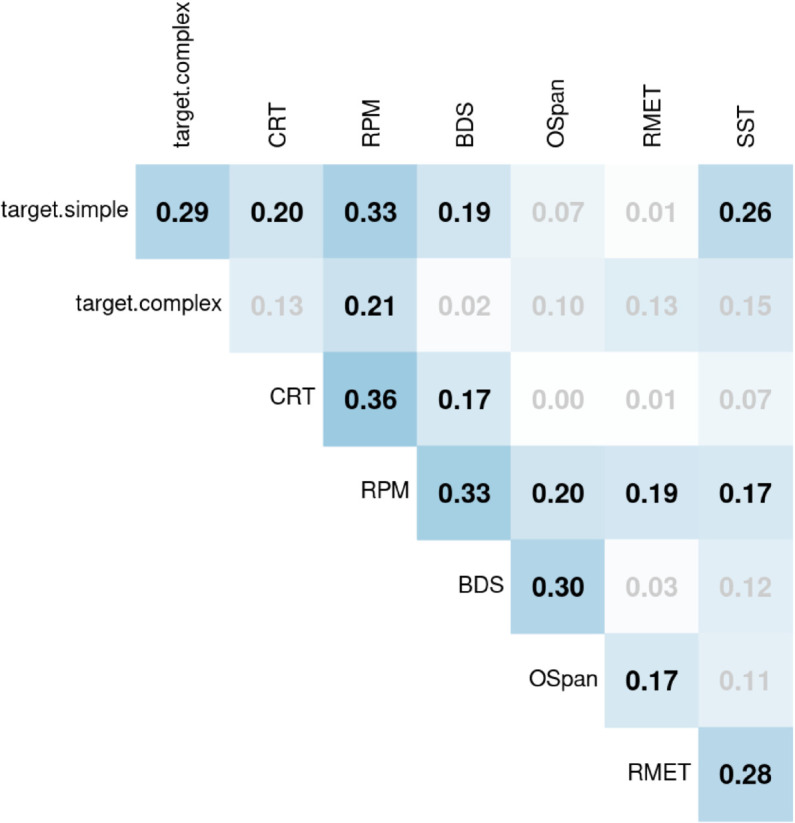
Correlations of the individual difference measures. Insignificant correlations are greyed out.

The summary statistics of the individual differences measures are reported in Table 5, and the correlation matrix, including the two conditions of the reference game, is included in Fig 6.

**Table 5:** Summary statistics for the individual difference measures (*N* = 167).

Measure	Mean	SD	Observed range	Possible range	Skewness	Kurtosis
OSpan	0.79	0.16	0.19-1	0-1	-1.33	4.69
BDS	4.75	1.51	2-8	0-8	0.34	2.39
RPM	5.38	2.14	0-9	0-10	-0.13	2.23
CRT	0.35	0.25	0-1	0-1	0.45	2.42
RMET	27.58	3.82	15-36	0-36	-0.59	3.44
SST	8.88	2.81	2-14	0-16	-0.23	2.32

The distributional statistics of the individual differences show that there is variability in all of the measures that none of the measures are at ceiling.

The correlations reported in the correlation matrix are corrected for multiple comparisons using FDR (False Discovery Rate).

The correlation matrix reveals that the measures which we selected to reflect the same underlying construct are moderately correlated with each other (CRT and RPM at *r* = 0.36, *p<*0.0001, BDS and OSpan at *r* = 0.30, *p* = 0.001, and RMET and SST at *r* = 0.28, *p* = 0.003).

Examining the correlation of the individual differences measures with average performance on the two reference game conditions (simple and complex) reveals a distinct pattern for the two conditions. While performance on the complex condition only correlates with RPM (*r* = 0.21, *p* = 0.02), the simple condition correlates positively with a larger number of measures: both measures of logical reasoning, CRT (*r* = 0.20, *p* = 0.03) and RPM (*r* = 0.33, *p* = 0.0001), as well as with BDS (*r* = 0.19, *p* = 0.03) and SST (*r* = 0.26, *p* = 0.002). When we conduct a regression analysis later, we include interaction terms of condition with individual difference measures to further statistically test this pattern.

The only other significant correlations are relatively weak correlations between CRT and BDS (*r* = 0.17, *p* = 0.046) and between OSpan and RMET (*r* = 0.17, *p* = 0.046), and between RPM and all other individual difference measures. The correlation between CRT and BDS is not surprising given that digit span tasks have been shown to have a linear positive relationship with general intelligence [55], which is related to reflective logical thinking which CRT taps into. The significant, though weak, correlation between OSpan and RMET is more surprising as we would not expect RMET to rely on working memory. This correlation may be attributed to the fact that the two tasks were both included in Session 2, and this correlation may simply be reflective of participants’ attention and disposition on that day. The fact that all cognitive tasks correlate significantly with RPM is not very surprising since all these tasks involve an element of reasoning and pattern recognition.

Since we collected two measures per construct (RPM and CRT for logical reasoning ability, OSpan and BDS for working memory, and RMET and SST for Theory of Mind), we conduct principal component analysis (PCA) in order to test whether these measures will indeed load onto three components representing these three constructs. We opted for PCA as opposed to CFA because some of the tasks which are supposed to tap into the same construct are not necessarily very similar or strongly correlated. For instance, while the Short Story Task and the Reading the Mind in the Eyes test are both said to measure Theory of Mind, SST involves interpreting intention from text and RMET involves emotion recognition, and they correlate at *r* = 0.28.

We conducted a Principal Component Analysis (PCA) with varimax rotation on the standardized individual differences measures (centered and scaled to a 0–1 range). Three principal components (PCs) were retained based on eigenvalues greater than 1; the remaining components had eigenvalues of 0.74 or lower. The selected PCs accounted for 23%, 23%, and 22% of the total variance, respectively; all together, they accounted for 68% of the variance. Factor loadings are presented in Table 6. The components align closely with the intended cognitive constructs: PC1 reflects logical reasoning ability (CRT and RPM), PC2 reflects Theory of Mind (RMET and SST), and PC3 reflects working memory (OSpan and BDS). In each case, the two measures which are thought to tap into that construct had loadings above 0.70, while the remaining measures loaded weakly (≤0.35, most below 0.20), indicating that the components represent their respective constructs with minimal overlap. We will therefore use the obtained factors as predictors of participants’ performance on the reference game.

**Table 6:** PCA factor loadings.

	PC1 (Logical reasoning)	PC2 (ToM)	PC3 (Memory)
CRT	**0.87**	-0.03	-0.07
RPM	**0.70**	0.23	0.33
RMET	-0.02	**0.82**	0.08
SST	0.14	**0.75**	0.04
OSpan	-0.12	0.17	**0.84**
BDS	0.35	-0.06	**0.73**

**Fig 7:**
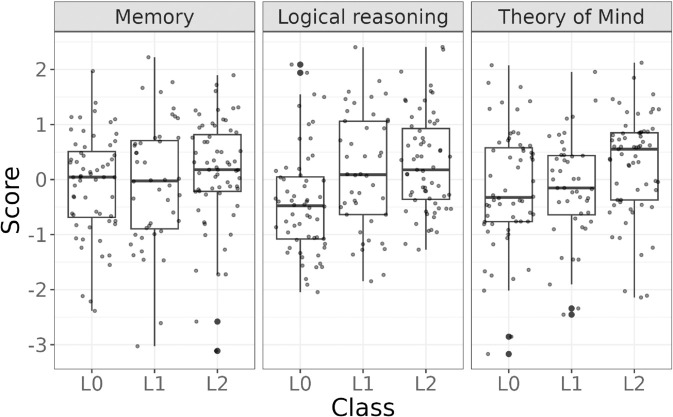
A boxplot showing the distribution of the three individual differences for each reasoning class identified by LPA. The y-axis represents the composite score for the corresponding individual difference obtained using PCA. Ordinal regression revealed the effect of logical reasoning and ToM but not of memory on reasoning class.

Before progressing to a continuous analysis, we look at the distribution of the three individual difference constructs for the three reasoning type clusters obtained by LPA from the previous section. Boxplots of the distributions of memory, logical reasoning and Theory of Mind for each of the three clusters identified by LPA are shown in Fig 7. While the distribution is fairly wide for all measures, we can see a general increasing trend from *L*_0_ to *L*_2_ for all three measures, where the mean memory, logical reasoning and ToM score for *L*_0_ is lower than for *L*_2_. This pattern appears clearest for logical reasoning and least clear for memory.

To verify these visually evident patterns, we conduct a simple ordinal regression using the *ordinal* package in R, with the assigned reasoning class (*L*_0_, *L*_1_ and *L*_2_) as the dependent variable and the three individual difference constructs as predictors. The model reveals a positive effect of logical reasoning (*β* = 0.58, *SE* = 0.15, *p<*0.001) and of Theory of Mind (*β* = 0.38, *SE* = 0.15, *p* = 0.01). The estimate for memory is positive but the effect does not reach significance (*β* = 0.17, *SE* = 0.15, *p* = 0.24).

Thus, belonging to a more sophisticated reasoning class is associated with better logical reasoning ability and Theory of Mind skills. It appears on Fig 7 that there may also be groupings between classes such that *L*_1_ and *L*_2_ are closer together in terms of logical reasoning than *L*_0_, and that *L*_0_ and *L*_1_ are closer to each other in terms of ToM than *L*_2_. However, we do not find statistical evidence for that distinction, as the Brant test indicated that neither predictor violated the proportional odds assumption (omnibus $\chi^{2}(3)$ = 3.00, *p* = 0.39).

The observed differences between the identified classes in terms of Theory of Mind are in line with the findings of Battaglini et al. [83], who used a data-driven approach to identify groups of responders in a metaphor interpretation task. One of the two groups they identified, which they term *mentalizers*, used more cognitive and affective terms in their metaphor interpretation, suggesting heavier ToM use. Their mentalizers group may correspond to the *L*_2_ class we identify, which is associated with higher ToM scores.

Next, we will conduct a full continuous analysis, which additionally includes the relationship between the individual differences and the two conditions in the reference game separately, as well as covariates and random effects.

#### Regression analysis with individual difference measures.

We again conduct Bayesian logistic regression. For this analysis, we keep only critical trials (simple and complex), since ambiguous trials were included in the original model only as a chance baseline to compare the complex condition and there is no theoretical reason to believe that individual differences should affect performance on the ambiguous trials. Trial correctness, a binary variable, is regressed onto condition (dummy-coded with complex as reference), trial number (mean-centered), participant age (mean-centered), target position (left, middle or right, dummy-coded with left as reference), message type (sum-coded, -1 = shape, 1 = color), the interaction of trial number and condition, main effects of individual differences (logical reasoning, memory and ToM) and interactions of individual differences with condition. Like before, the random effects structure included per-participant and per-item random intercepts and per-participant random slopes for condition, message type and trial number.

For all effects, very wide weakly informative priors centered at 0 were set. The full model equation is included in S2 Appendix. We ran four chains of 6000 iterations each, with the first 1000 iterations of each chain discarded as warmup. The regression results are reported in Table 2.

Main effect of condition persists: participants performed better on simple trials than on complex trials ($\hat{\beta }$ = 0.97, 95% CrI = [0.60, 1.34]). There was again a small positive effect of trial number ($\hat{\beta }$ = 0.01, 95% CrI = [0.01, 0.02]). Like in the first model, participants were more likely to choose the target if it was in the middle compared to the left ($\hat{\beta }$ = 0.53, 95% CrI = [0.33, 0.74]) and on the left compared to the right ($\hat{\beta }$ = -0.21, 95% CrI = [-0.40, -0.03]). Unlike the model without individual differences, this model does not show an effect of message type ($\hat{\beta }$ = -0.10, 95% CrI = [-0.23, 0.02]) but it does show a small negative effect of age, such that older participants are less likely to respond correctly ($\hat{\beta }$ = -0.02, 95% CrI = [-0.03, -0.01]).

In terms of individual differences, there is a positive main effect of logical reasoning ($\hat{\beta }$ = 0.22, 95% CrI = [0.06, 0.38]) and ToM ($\hat{\beta }$ = 0.26, 95% CrI = [0.10, 0.42]), meaning that participants with higher logical reasoning and ToM selected the target more often. There is no main effect of memory ($\hat{\beta }$ = 0.06, 95% CrI = [-0.10, 0.22]).

**Fig 8:**
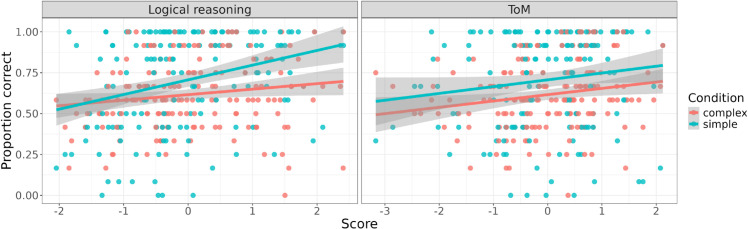
Proportion of correct responses of individual participants in the two implicature conditions as a function of logical reasoning and ToM.

There is also a positive interaction of logical reasoning with condition, whereby the effect of logical reasoning is stronger in the simple condition ($\hat{\beta }$ = 0.45, 95% CrI = [0.13, 0.78]), but no interaction of memory or ToM with condition. Fig 8 visualizes the effects of logical reasoning and ToM in the two implicature conditions.

## Discussion

In this study, we conducted the pragmatic reference game task from F&D on a much larger sample and systematically investigated substantial individual variability in performance, relating it to individual differences in cognitive traits.

We identified groups of participants using Latent Profile Analysis and found, consistent with F&D, that they approximately correspond to the predictions of the three idealized reasoning types formalized by three RSA models of different reasoning depth – literal listener *L*_0_, pragmatic listener reasoning about a literal speaker *L*_1_, and Gricean listener *L*_2_. Unlike F&D, for whom only a small proportion of their participants fell into the *L*_2_ class, in our data, that is the largest identified class. This could be related to the difference in the stimuli – whereas F&D used images of mosters, robots and accessories for their study, we opted for the more abstract shapes and colors since Mayn & Demberg [14] found that the original stimuli were more susceptible to biases. It could also be that in F&D’s sample there were so few participants who could solve the task due to sampling chance, as their sample size was significantly smaller than ours (51 vs. 254 participants for the main task). Additionally, at the end of the experiment, we asked participants to report their strategy for one simple and one complex implicature trial as a way to gain additional insight into how participants performed the task and how strategy relates to performance. We found that overall, there was a decent degree of alignment between participants’ performance and their reported strategy: most participants assigned to the literal *L*_0_ class reported guessing on both types of implicature trials and most participants assigned to the Gricean *L*_2_ class reported applying correct counterfactual reasoning for both trial types. However, there was some misalignment, presumably related at least in part to lucky guessing (i.e., having high performance on a condition despite applying a guessing strategy) or to figuring out how to solve the critical trials during the task. In the regression model, we find a small effect of trial number, suggesting that some participants may have learned how to solve the implicatures during the task. Finally, there were two groups of participants who could be identified based on annotations of their reported strategy who used different strategy which led them to have very low performance: one group of participants misunderstood the meaning of available messages, and the other applied inverse reasoning, e.g., interpreting the message “red" to mean “the only *non-red* object". Those participants were excluded from the LPA and regression analyses since the relationship between strategy and performance is different for them than for the rest of the participants.

Having established that there is sizeable variability in performance which reflects application of different strategies, we investigated the source of that variability by relating it to measures of cognitive traits. In addition to the reference game, we collected six individual differences measures belonging to three constructs: logical reasoning (Cognitive Reflection Test and Raven’s Progressive Matrices), working memory (Operation Span and Backward Digit Span) and Theory of Mind (Reading the Mind in the Eyes and the Short Story Task). We then conducted a principal component analysis and found that the tasks indeed loaded into the corresponding components. We observed an effect of logical reasoning: participants with better logical reasoning skills responded more pragmatically. This effect was more pronounced for simple implicature trials compared to complex trials. We also found an effect of Theory of Mind, whereby participants with better mentalizing skills performed better on the reference game. We did not find an effect of working memory.

Notably, the observed effects are additive in nature: i.e., the effect of logical reasoning persists after WMC and Theory of Mind are controlled for, and the same holds for the effect of Theory of Mind. This is important to stress since the three cognitive constructs we investigated are not entirely independent – working memory and fluid intelligence, which is related to our logical reasoning component, are known to correlate and to draw on higher-level executive processes, such as attention and disengagement [84,85]. Theory of Mind has also been shown to be related to intelligence and executive function: previous work has observed a weak positive relationship between RMET and SST and fluid intelligence measures (RPM, as well as standardized tests), suggesting that mentalizing as measured by these tasks draws on general cognitive abilities [86,87]. Our findings suggest that logical reasoning and mentalizing have distinct contributions to pragmatic processing.

One could ask whether the significant effect of logical reasoning ability on reasoning strategy might be related to the fact that the reference game is a nonlinguistic, somewhat puzzle-like task and whether this effect of logical reasoning would generalize to linguistic pragmatic tasks. Ryzhova et al. [6] found an effect of logical reasoning, also measured using Raven’s Progressive Matrices and Cognitive Reflection Test, for a linguistic atypicality inference task, whereby better reasoners were more likely to interpret a redundant utterance pragmatically. This suggests that logical reasoning may modulate pragmatic performance beyond nonlinguistic reference games. However, Heyman & Schaeken [3] found that performance on the Cognitive Reflection Test did not predict the rate of scalar implicature derivation for their participants. It is therefore possible that logical reasoning is needed for novel settings, whereas for more conventionalized expressions the reasoning may be amortized [88]. If this were the case, then there would more likely be an effect of logical reasoning for novel metaphors than conventionalized metaphors, for instance. The effect of logical reasoning ability on other pragmatic tasks could be investigated in future work.

Interestingly, the observed effect of logical reasoning was stronger in the simple implicature condition than in the complex implicature condition. Given that performance was generally lower on the complex condition, it is possible that the complex condition was too complex for some people to solve even despite good reasoning skills, whereas in the simple condition, better reasoners were more likely to select the right approach to the task.

The fact that an effect of Theory of Mind emerges suggests that the framing of the reference game as a communication game, where participants are told that they are interpreting messages sent by a participant of a previous experiment, was sufficient to induce perspective-taking and reasoning about why the speaker might have said what they did. Notably, the tasks we used for measuring Theory of Mind, Reading the Mind in the Eyes and the Short Story Task, are both quite different from the reference game, and were conducted in a different session. The fact that an effect still emerges suggests that there is an element of mentalizing about the speaker to the pragmatic reasoning involved in the reference game.

We did not observe an effect of working memory capacity (WMC), which is not particularly surprising given mixed evidence of the role of WMC in other pragmatic phenomena. Since we did not observe a direct effect of memory, we hypothesized that an indirect effect of memory might emerge if working memory is employed for supporting logical reasoning or Theory of Mind computations – that is what Fairchild & Papafragou [5] observed for scalar implicatures, metaphors and indirect requests. Since the principal components which we used as predictors are decorrelated, in an additional analysis, we computed composite scores of each individual difference construct by summing up z-scores for the corresponding tasks and used those as predictors instead. We then compared a full model with all three individual difference predictors and models with one or both of logical reasoning and Theory of Mind taken out to see if an effect of memory emerges, which would provide indirect evidence for a possible role of WMC. In the full model with composite scores, exactly the same effects were observed as in the model with principal components which we reported in the paper. Moreover, even in the model where both logical reasoning and Theory of Mind were taken out, the credible interval for the effect of WMC contained zero, meaning that we do not observe even indirect evidence for the role of working memory on this task.

We opted for using principal components for our main analysis since individual tasks may be more prone to task impurity [89]. However, given the observation we made when examining correlations of the individual differences with the two reference game conditions – that only Raven’s Progressive Matrices correlated significantly with the complex condition while a larger number of measures (both CRT and RPM, as well as SST and BDS) correlated with the simple condition, we conducted an additional analysis where we included the logical reasoning and ToM measures individually as predictors instead of the principal components. A further motivation is that the individual difference measures we selected for each construct tap into distinct constructs in addition to the shared construct: CRT has been argued to measure reflective thinking [45], whereas RPM is a measure of fluid intelligence and abstract reasoning; RMET is thought to tap emotion recognition [77], while SST may be more representative of ability to reason about others’ mental states. The model estimates are reported in S3 Appendix. In the model with individual predictors, the main effect of CRT and interactions of condition with RPM and SST come out; there is no effect of RMET. This may suggest that reflective thinking and abstract reasoning both make a distinct contribution to pragmatic processing, and that cognitive Theory of Mind, i.e., ability to imagine other people’s mental states and thoughts, is more relevant to this task than emotion recognition. That would not be surprising since this a pragmatic reasoning task in which emotions should not play a large role.

There is ample evidence that different pragmatic tasks vary in the degree to which they require logical reasoning, working memory and mentalizing (see e.g. [90]) and that a more differentiated, task-specific view of the role of individual differences in Pragmatics is necessary – indeed, the fact that studies with different pragmatic tasks yield different findings is evidence of that. In addition, it has been argued that the interpretive strategy which is employed on a given task – and different strategies may tax cognitive resources to different extents – is also dependent on the comprehender’s profile [91]. Our findings that different individuals recruit different strategies for solving the reference game and that those strategies are predicted by Theory of Mind and logical reasoning align with that view.

We would like to briefly discuss where the difference in complexity between simple and complex implicature trials might stem from. As we noted earlier, the *L*_1_ model is able to solve the simple trials better than the complex ones, and, consistent with that model, the majority of our participants have better performance on the simple trials (Fig 3). Additionally, we observed a stronger effect of logical reasoning ability for simple implicature trials. Taken literally, the *L*_1_ model’s ability to solve simple trials is based on reasoning not about the speaker but about the available messages: it assumes that the speaker is literal, and the listener reasons that since there is only one way to refer to the target and two ways to refer to the competitor, and the probability mass is split between them, the message is twice as likely to be referring to the target. In an earlier study [17], we showed that when participants were presented with an explicitly literal speaker, they failed to reason about alternative messages and behaved as *L*_0_s and not as *L*_1_s themselves, suggesting that reasoning about the distribution of available signals comes less naturally to people than reasoning about the speaker’s intent. Indeed, when we examine participants’ reported strategies for the simple condition in this experiment, they also overwhelmingly refer to the speaker’s intent – that they would have used a different message if they had meant a different referent – as opposed to the distribution of available messages. This suggests that *L*_1_ grounds the complexity of simple inferences themselves rather than accurately describing the reasoning process used by participants to derive them. A processing-level modeling approach may be needed to construct a plausible model of how participants solve simple vs. complex inferences (see Duff et al. [92] for a recent proposal of such an approach in ACT-R). One observation is that, in the simple condition, it is sufficient to pay attention to the target and the competitor to solve the implicature since the distractor is completely irrelevant to the computations. In the complex condition, on the other hand, the distractor needs to be taken into account when deriving the inference, which means that reasoning involving more objects need to be performed.

Reference games are widely used for testing predictions of Rational Speech Act models, with the claim that this minimal signaling game captures some important properties of pragmatic communication using language “in the wild" [15]. However, reference games are in many ways unlike communication using language, and it is a warranted question to what extent findings in this setting generalize to language use. The effect of Theory of Mind which we observe suggests that reasoning about the speaker’s perspective which is assumed to be an essential part of pragmatics of language is at least in part preserved in the reference game setting. Also, exactly because reference games have been so widely used in probabilistic pragmatics, it is valuable to better understand their properties and their relationship to individual differences in cognition. Insights gained from this individual difference analysis can be useful for developing processing-level models of pragmatic reasoning.

## Supporting information

S1 Appendix Regression model output with additional exclusions based on Raven’s matrices. (PDF)

S2 Appendix Regression model priors. (PDF)

S3 Appendix Regression model output with individual measures instead of principal components. (PDF)
