# Supplementary material for: Sources of individual variability in a pragmatic reference game: Effects of logical reasoning and Theory of Mind
Source: PLoS One. 2026 Feb 19;21(2):e0339899. doi: 10.1371/journal.pone.0339899 (PMC12919809; doi:10.1371/journal.pone.0339899)
Supplement: S1 Appendix — (PDF) [file pone.0339899.s001.pdf]

S1 Appendix. Regression model output with additional exclusions based on Raven's matrices

The table below shows the output of a regression model which additionally excludes 8 people who responded "Yes" or "Not sure" to the question whether they recall seeing any of the questions on the Raven's matrices test before, resulting in a sample size of 159 participants.

The effect size estimates and credible intervals are very similar to those reported in the main analysis, where these participants were included. The interpretation of the results remains unchanged.

	**Model with Raven's exclusions (*N*=159)**	
**Effect**	**Estimate**	**95% CrI**
Intercept	**0.45**	[0.22, 0.68]
condition1 (simple vs. complex)	**1.01**	[0.62, 1.42]
trial number	**0.01**	[0.00, 0.02]
msgtype (color vs. shape)	-0.09	[-0.22, 0.04]
targetpos (middle vs. left)	**0.54**	[0.34, 0.75]
targetpos (right vs. left)	**-0.23**	[-0.42, -0.03]
condition1 : trial	**-0.01**	[-0.02, -0.00]
logical reasoning	**0.21**	[0.04, 0.39]
memory	0.05	[-0.12, 0.21]
ToM	**0.20**	[0.03, 0.37]
condition1 : reasoning	**0.41**	[0.06, 0.77]
condition1 : memory	0.23	[-0.11, 0.58]
condition1 : ToM	0.18	[-0.17, 0.52]

Table. Output of the regression model with individual differences, where participants who reported being familiar with any of the questions on Raven's matrices were excluded.
