# Supplementary material for: Sources of individual variability in a pragmatic reference game: Effects of logical reasoning and Theory of Mind
Source: PLoS One. 2026 Feb 19;21(2):e0339899. doi: 10.1371/journal.pone.0339899 (PMC12919809; doi:10.1371/journal.pone.0339899)
Supplement: S2 Appendix — (PDF) [file pone.0339899.s002.pdf]

S2 Appendix. Regression model priors

Model without individual differences

We fit the following Bayesian logistic regression model to the data using the *brms* package in R:

$$\begin{aligned} binaryCorrectness \sim condition + trialNumber + condition * trialNumber + \\ targetPos + msgType + (1 + condition + msgType \\ + trialNumber|participantID) + (1|itemID) \end{aligned}$$

For all effects, we set wide weakly informative priors in order to avoid biasing the model. Below we list the priors with a brief rationale.

- Fixed effects:
  - Prior: Normal(0, 2).
  - Rationale: For all main effects and interactions we set a wide prior centered at 0. The wide standard deviation of 2 was chosen after examining the effect sizes in priors work in similar paradigms (see Table 3 in the article). This wide prior is chosen to be conservative and allow for flexibility in estimating effect sizes.
- Standard deviation of random effects for *participantId* and *itemId*
  - Prior: Student_t(3, 0, 1)
  - Rationale: Student's-t distribution with 3 degrees of freedom, centered at 0 with a scale of 1 was chosen to be robust to outliers and to capture for potential variability between participants and items beyond that accounted for by fixed effects.
- Correlation structure
  - Prior: LKJ(2)
  - Rationale: We set a weakly informative prior, allowing for moderate correlation of random effects.

Model with individual differences

This model includes the same effects as the model without the individual differences. It additionally includes main effects of the three individual differences, as well as their interactions with condition:

$$\begin{aligned} binaryCorrectness \sim condition + trialNumber + condition * trialNumber + \\ targetPos + msgType + condition * reasoning + condition * WMC + condition * ToM + \\ (1 + condition + msgType + trialNumber|participantID) + (1|itemID) \end{aligned}$$

The same wide regularizing priors as for the model without individual differences (discussed in the previous subsection) were set for all effects.
