# Supplementary material for: Sources of individual variability in a pragmatic reference game: Effects of logical reasoning and Theory of Mind
Source: PLoS One. 2026 Feb 19;21(2):e0339899. doi: 10.1371/journal.pone.0339899 (PMC12919809; doi:10.1371/journal.pone.0339899)
Supplement: S3 Appendix — (PDF) [file pone.0339899.s003.pdf]

# S3 Appendix. Regression model output with individual measures instead of principal components

The table below shows the output of a regression model where the individual logical reasoning and ToM measures are used as predictors instead of the principal components ($N$=167).

Interestingly, when individual difference measures are included individually, only CRT has a main effect ($\hat{\beta}$=0.69, 95% CrI = [0.00, 1.38]). There are also noteworthy interactions between condition and RPM ($\hat{\beta}$=1.53, 95% CrI = [0.19, 2.89]) and SST ($\hat{\beta}$=1.46, 95% CrI = [0.15, 2.80]). This might suggest that the relationship between pragmatic processing in the reference game is primarily driven by fluid intelligence or pattern recognition as opposed to reflective thinking, and by cognitive as opposed to affective Theory of Mind.

| | **Model with ID measures instead of PCs (*N*=167)** | |
|---|---|---|
| **Effect** | **Estimate** | **95% CrI** |
| Intercept | -0.88 | [-1.55, 1.20] |
| condition1 (simple vs. complex) | -0.32 | [-1.53, 0.88] |
| trial number | **0.01** | [0.01, 0.02] |
| participant age | **-0.02** | [-0.04, 0.01] |
| msgtype (color vs. shape) | -0.10 | [-0.22, 0.02] |
| targetpos (middle vs. left) | **0.53** | [0.33, 0.74] |
| targetpos (right vs. left) | **-0.21** | [-0.40, -0.03] |
| condition1 : trial | -0.01 | [-0.02, 0.00] |
| CRT | **0.69** | [0.00, 1.38] |
| RPM | 0.45 | [-0.29, 1.18] |
| RMET | 0.82 | [-0.08, 1.73] |
| SST | 0.58 | [-0.11, 1.27] |
| condition1 : CRT | 0.42 | [-0.85, 1.70] |
| condition1 : RPM | **1.53** | [0.19, 2.89] |
| condition1 : RMET | -1.02 | [-2.64, 0.61] |
| condition1 : SST | **1.46** | [0.15, 2.80] |

**Table.** Output of the regression model with individual differences in logical reasoning and ToM without principal components.

The figure below visualizes the effects of RPM and SST in the two implicature conditions.

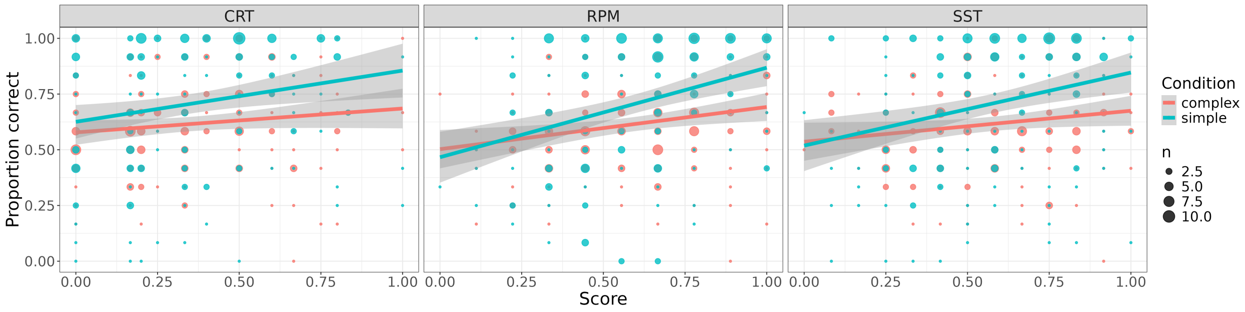


**Figure.** Proportion of correct responses of individual participants in the two implicature conditions as a function of CRT, RPM and SST.
